# Cows painted with zebra-like striping can avoid biting fly attack

**DOI:** 10.1371/journal.pone.0223447

**Published:** 2019-10-03

**Authors:** Tomoki Kojima, Kazato Oishi, Yasushi Matsubara, Yuki Uchiyama, Yoshihiko Fukushima, Naoto Aoki, Say Sato, Tatsuaki Masuda, Junichi Ueda, Hiroyuki Hirooka, Katsutoshi Kino

**Affiliations:** 1 Animal Husbandry Division, Aichi Agricultural Research Center, Nagakute, Aichi, Japan; 2 Laboratory of Animal Husbandry Resources, Division of Applied Biosciences, Graduate School of Agriculture, Kyoto University, Kyoto, Japan; 3 Aichi Veterinary Association, Nagoya, Aichi, Japan; University of Illinois, UNITED STATES

## Abstract

Experimental and comparative studies suggest that the striped coats of zebras can prevent biting fly attacks. Biting flies are serious pests of livestock that cause economic losses in animal production. We hypothesized that cows painted with black and white stripes on their body could avoid biting fly attacks and show fewer fly-repelling behaviors. Six Japanese Black cows were assigned to treatments using a 3 × 3 Latin-square design. The treatments were black-and-white painted stripes, black painted stripes, and no stripes (all-black body surface). Recorded fly-repelling behaviors were head throw, ear beat, leg stamp, skin twitch, and tail flick. Photo images of the right side of each cow were taken using a commercial digital camera after every observation and biting flies on the body and each leg were counted from the photo images. Here we show that the numbers of biting flies on Japanese Black cows painted with black-and-white stripes were significantly lower than those on non-painted cows and cows painted only with black stripes. The frequencies of fly-repelling behaviors in cows painted with black-and-white stripes were also lower than those in the non-painted and black-striped cows. These results thus suggest that painting black-and-white stripes on livestock such as cattle can prevent biting fly attacks and provide an alternative method of defending livestock against biting flies without using pesticides in animal production, thereby proposing a solution for the problem of pesticide resistance in the environment.

## Introduction

Zebras have a bold black-and-white striped pattern on their body surface. Many functional hypotheses on the striped pattern of zebras were generated in scientific researches, such as camouflage, confusion of predators, signaling to conspecifics, thermoregulation, and avoidance of biting flies [1]. Among these hypotheses, several studies now indicate that preventing attack by biting flies is the function of zebra stripes. For example, Caro *et al*. [2] showed that the phylogenetic distribution of body stripes is associated with tabanid fly distributions at the species and subspecies level. Additionally, Egri *et al*. [3] experimentally showed that tabanids avoid landing on black-and-white surfaces, such as trays, boards, balls, and buckets. Moreover, Caro *et al*. [4] demonstrated that tabanids flies are far less likely to land on striped cloth coats than on black or white coats when placed on horses. In contrast, the other hypotheses such as camouflage, confusion of predators, social interaction, and heat management have not been supported by researchers [1, 5–9].

Research further shows that biting flies eschew landing on white [10], striped [3, 11], and spotted surfaces [12]. Stripes narrower than a critical width (approximately 5 cm) and spots smaller than a threshold size (diameter approximately 10 cm) effectively prevent biting flies from landing and these surfaces attract fewer biting flies than white surfaces [3, 12].

Biting flies are serious pests of livestock [13–15] and previous studies have reported that biting flies affect the behavior of cattle and cause economic losses [16–18]. In fact, biting flies reduce grazing, feeding, and bedding down time of cattle and increase fly-repelling behaviors (e.g. head throwing, foot stamping, skin twitching, and tail flicking) and bunching behaviors of cattle [15, 18]. Bunching increases heat stress and risk of injury as animals jostle for a better position to avoid biting flies, and this can reduce weight gains in feedlot beef cattle and milk yield in dairy cows [15, 17, 18].

We hypothesized that cows painted with black-and-white stripes on their body could avoid biting fly attacks and decrease their fly-repelling behaviors. This may be an alternative environmentally friendly practical method of controlling biting flies without the use of pesticides in animal production. Therefore, this study investigated whether painting zebra-like stripes on cattle reduces the number of biting flies landing on them and reduces the frequency of their fly-repelling behaviors. To our knowledge, this is the first study to evaluate the effect of zebra-like stripes painting on fly attacks on live animals.

## Materials and methods

All procedures were approved by the Animal Use and Care Committee of the Aichi Agricultural Research Center (29–1). The experiment was conducted in August and September 2017 and in October 2018 on 1 ha of natural pasture at Aichi Agricultural Research Center in Nagakute-city, Japan (35°16ʹ N, 137°07ʹ E). Mean temperatures on the experimental days were 30.4 ± 1.7°C in 2017 and 20.1 ± 2.2°C in 2018 between 10:00–12:00 and 31.1 ± 1.8°C in 2017 and 20.4 ± 1.4°C in 2018 between 13:00–15:00. Six pregnant Japanese Black cows with average body weight of 481.3 ± 46.9 kg were used. The cows were familiar with the study area and had continuously grazed in the pasture during the grazing season (early April to late November in 2017 and 2018). The cows were fed with concentrate (0.5 kg/head/day) at 10:00 daily. Water and mineral were available *ad libitum*.

**Fig 1:**
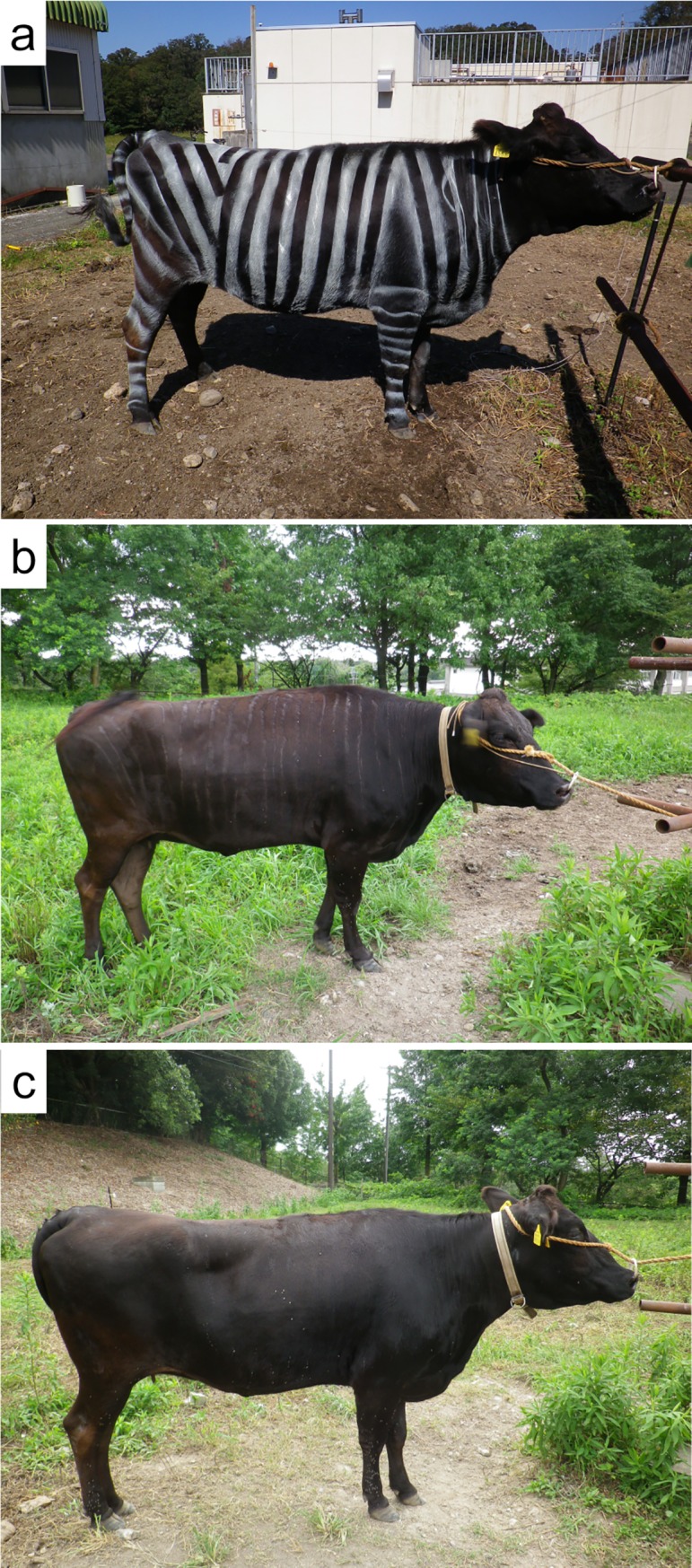
Examples of experimental cows with/without painted stripes. (a) Black-and-white striped cattle (B&W) with white lacquer, (b) Black-striped cattle (B) with black lacquer, (c) No stripe cattle (CONT).

The cows were assigned to treatments using a 3 × 3 Latin-square design. The treatments consisted of black-and-white painted stripes (B&W), black painted stripes (B), and no stripes (CONT) as a control (Fig 1). Since Japanese Black cows are usually all black (including the cows used in this study), we used commercial waterborne white lacquers to create the B&W treatment and commercial waterborne black lacquers to create the B treatment. Striping by the waterborne lacquers (Color Spray BASIC, NIPPONPAINT Co., Ltd, Tokyo, Japan) fades easily (in a few days), which enabled us to assign the cows to the other treatments. The painted stripes were drawn freehand in width of approximately 4–5 cm and were painted on the morning of every observation day. Painting stripes on cows required approximately 5 minutes/individual.

Three of the six cows were used in August and September 2017 and the other three in October 2018. The same experimental design was adopted in both years but different animals were used. The experiment lasted for 9 days, and 3 consecutive days were allocated for a period for one treatment. Each cow experienced all three treatments (B&W, B, or CONT) in three periods and there was no cow assigned to the same treatment as the other two cows in a period.

**Fig 2:**
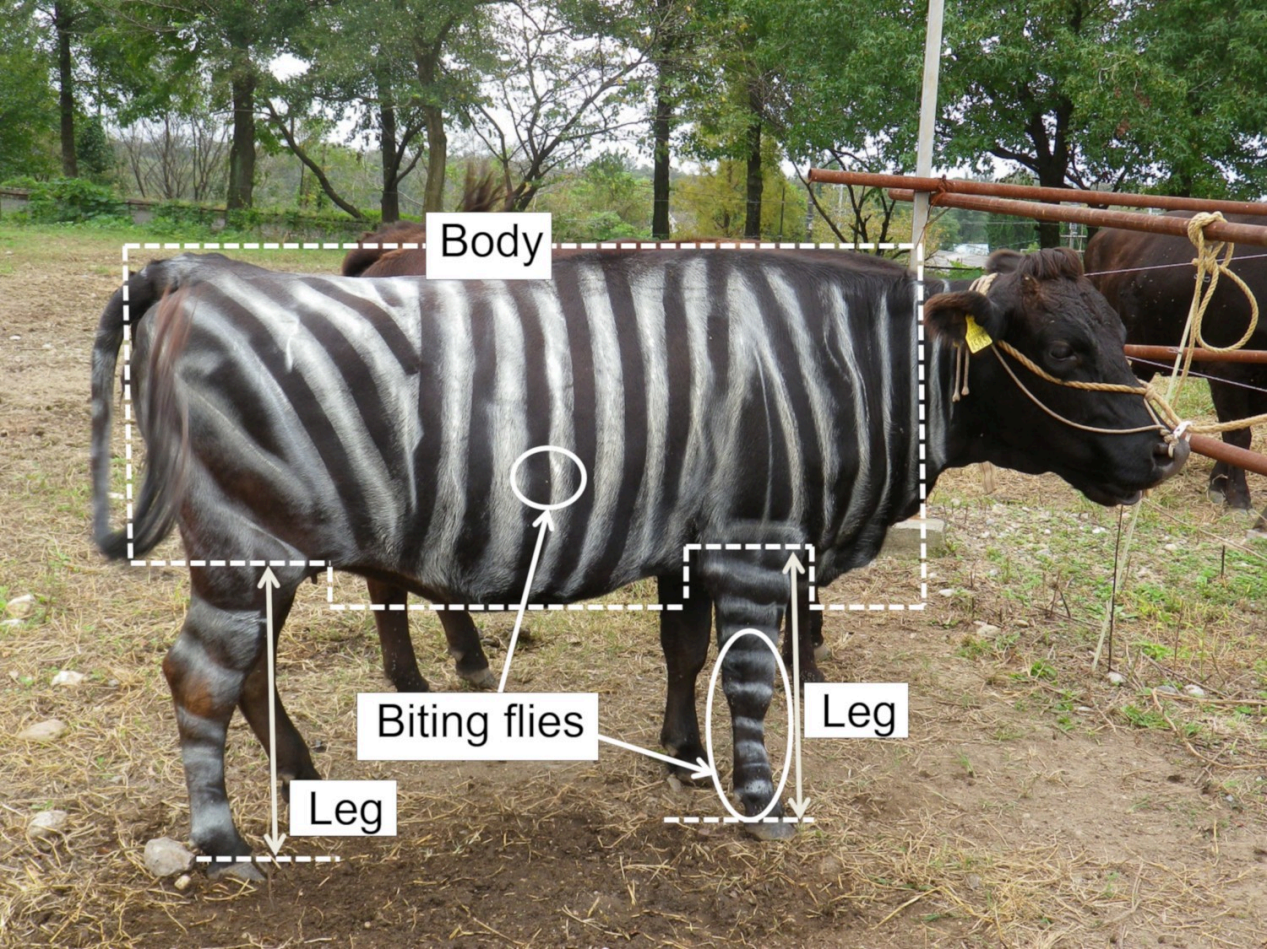
Example of the area of legs and body used to count biting flies on cows.

The cows were arranged side-by-side and tied with a rope attached to a stake in the ground that was long enough to enable them to move their heads freely. Each cow was observed twice a day (am/pm) and a total of six observations were obtained for each cow and treatment. The order of observations of each cow was randomized for each observation. The morning observations started 30 minutes after painting to freshen the air around the cow and allow the odor of paint to dissipate. In accordance with Eicher *et al*. [19], we used a 1-min interval instantaneous scan sampling technique for 30 min; therefore, each cow was observed for 30 instantaneous scans on the minute during 30 min twice daily for each period. Using this technique, one observer recorded the fly-repelling behaviors of each cow throughout the experimental period. Recorded fly-repelling behaviors were head throw, ear beat, leg stamp, skin twitch, and tail flick. The right sides of all three cows were observed simultaneously in sunlight or shade for each observation and photo images of the right side of each cow were taken after every 30-min observation using a commercial digital camera (PENTAX Optio RZ10). Biting flies on the body and each leg were counted from the photo images (Fig 2). The resolution of the images was 14 M pixels (4288 × 3216 pixels).

Moreover, thin, blue, plastic boards (T-trap, TSET Ltd, Aichi, Japan) (40 × 40 cm) were covered with transparent, odorless, and colorless glue and placed on the ground to trap and identify the species of biting flies around the experimental cows.

Statistical analysis was carried out in accordance with Kaps and Lamberson [20]. Data were analyzed using mixed linear models using the MIXED procedure in SAS (SAS Inst. Inc., Cary, NC). Treatments (B&W, B, and CONT) were analyzed as the fixed effect and Latin-square (2017 and 2018), period, and animals nested within the Latin-square were included as random effects. Multiple comparisons within the fixed effect were carried out using Tukey’s test. Dependent variables were the numbers of biting flies on legs, body, and the sum of biting flies on legs and body and the frequencies of head throws, ear beats, leg stamps, skin twitches, and tail flicks, and the total of these five behaviors (total behaviors).

## Results

**Fig 3:**
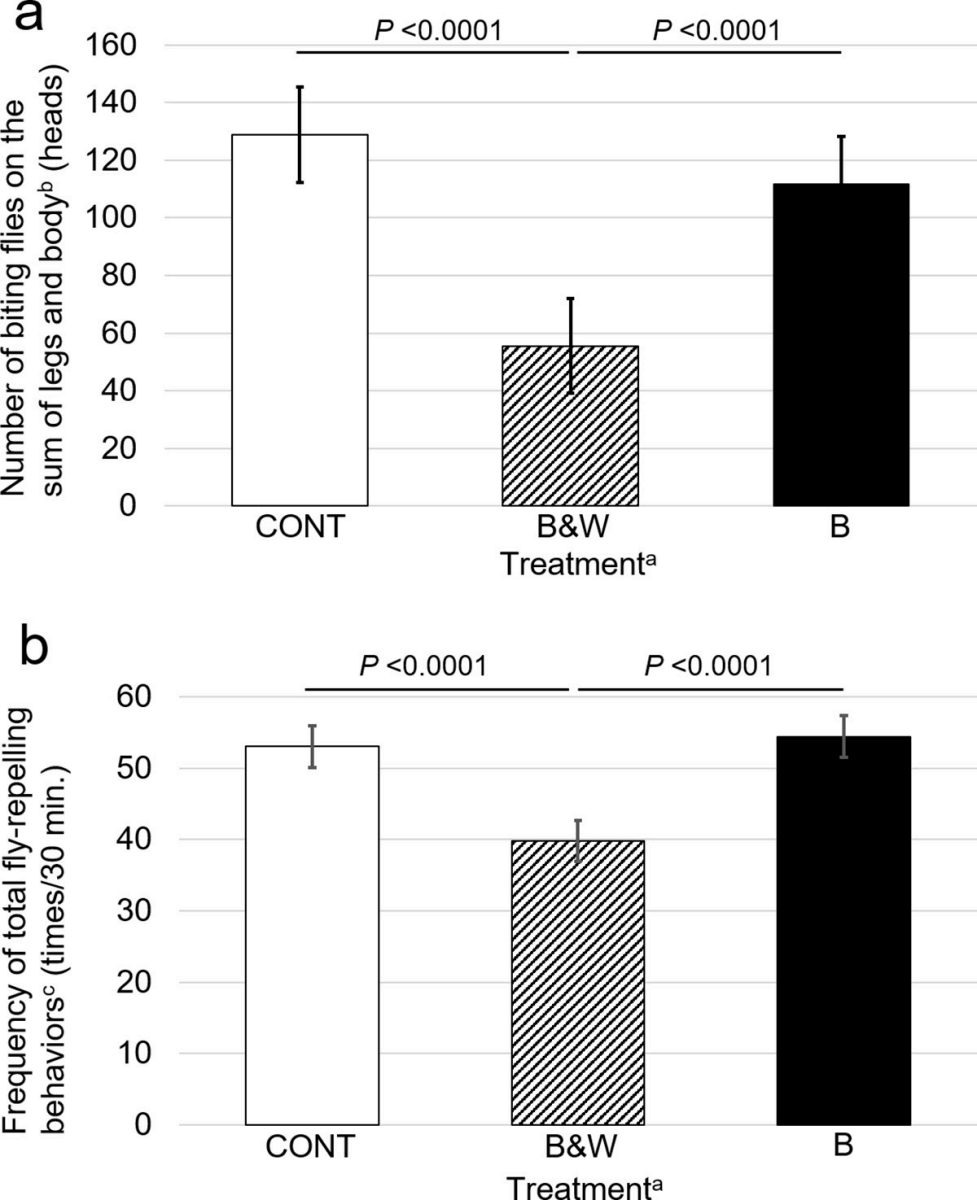
**Number of biting flies on legs and body (a) and the frequency of total fly-repelling behaviors (b) of the experimental cows.** ^a^CONT: no stripe cattle, B&W: black-and-white striped cattle with white lacquer, which indicates “striped-cattle” indicates “striped-horse (zebra)” in Japanese), B: black-striped cattle with black lacquer. ^b^Biting flies trapped by the sticky plastic boards were mainly stable flies (*Stomoxys calcitrans*), a few horn flies (*Haematobia irritans*), and horse flies (*Tabanus sapporoensis*). These flies are popular in Japan [21–24]. The relative abundance of these biting flies were *Stomoxys calcitrans* (77.9%), *Haematobia irritans* (21.5%), and *Tabanus sapporoensis* (0.5%). ^c^Head throws, ear beats, leg stamps, skin twitches and tail flicks.

The effects of black-and-white stripe painting (B&W), black stripe painting (B), and no painting (CONT) on biting fly attacks on Japanese Black cows with an all-black body color are presented in Fig 3 and Table 1 (treatments are shown in Fig 1). The total numbers of biting flies on legs, body, and the sum of legs and body for B&W cows were almost half those on CONT and B cows (*p* <0.0001 and *p* <0.001, respectively). In contrast, no significant differences in the number of biting flies were observed between CONT and B.

**Table 1:** Numbers of biting flies on the experimental cows and the frequencies of fly-repelling behaviors.

		Treatment^a^				Probabilities		
		CONT	B&W	B	s.e.	CONT VS B&W	CONT VS B	B&W VS B
Numbers of biting flies (heads)^b^								
	On legs^c^	86.7	40.2	73.1	11.1	<0.0001	0.12	<0.0001
	On body^c^	42.1	15.3	38.6	8.0	<0.0001	0.82	<0.001
Frequencies of fly-repelling								
behaviors (times/30 min.)								
	Head throws	1.94	0.72	1.89	0.72	<0.001	0.63	<0.05
	Ear beats	14.8	9.0	14.1	1.6	<0.0001	0.66	<0.0001
	Leg stamps	7.9	5.1	8.3	1.3	<0.001	0.83	<0.0001
	Skin twitches	2.36	3.58	2.44	1.22	<0.05	0.98	<0.05
	Tail flicks	27.0	21.2	27.4	1.0	<0.0001	0.92	<0.0001

^a^ CONT: no stripe cattle, B&W: black-and-white striped cattle with white lacquer, which indicates “striped-cattle” indicates “striped-horse (zebra)” in Japanese), B: black-striped cattle with black lacquer.

^b^ Biting flies trapped by the sticky plastic boards were mainly stable flies (*Stomoxys calcitrans*), a few horn flies (*Haematobia irritans*), and horse flies (*Tabanus sapporoensis*). These flies are popular in Japan [21–24]. The relative abundance of these biting flies were *Stomoxys calcitrans* (77.9%), *Haematobia irritans* (21.5%), and *Tabanus sapporoensis* (0.5%).

^c^ See Fig 2 in Text.

The effects of the treatments on fly-repelling behaviors are also shown in Table 1. For the B&W cows, 39.8 fly-repelling behaviors per 30 min were observed, which was significantly lower (*p* <0.0001; about 20%) than those observed in CONT (53.0 fly-repelling behaviors) and B (54.4 fly-repelling behaviors) cows. The lower number of fly-repelling behaviors in B&W cows was assumed to be because of the fewer numbers of biting flies on the legs and body. The frequencies of head throws, ear beats, leg stamps, and tail flicks of B&W were also significantly lower than those of CONT and B (*p* <0.05) cows, whereas the frequency of skin twitches of B&W was significantly higher than that of CONT and B (*p* <0.05) cows.

## Discussion

Biting flies are the most damaging arthropod pests of cattle worldwide and the economic impact of biting flies on the United States cattle production was estimated at $2,211 million per year [18]. Because of the economic loss associated with this pest, cattle owners have primarily used insecticidal control measures [25]. However, insects often evolve resistance to a new pesticide within about a decade after its introduction [26]. Hogsette *et al*. [27] demonstrated that horn flies soon became resistant to some pesticides and, therefore, pesticide-impregnated ear tags were no longer recommended for horn fly management programs in some parts of the United States. In the present study, black-and-white stripes decreased the numbers of biting flies on cattle and the frequencies of cow fly-repelling behaviors except skin twitches. Mullens *et al*. [15] pointed that fly-repelling behaviors could be divided into more frequent and less energy-intensive acts (skin twitches and tail flicks) or less frequent and more energy-intensive acts (head throws and foot stamps), and thereby skin twitches with low energy costs tended to occur in cases of low fly loads. In the present study, black-and-white painted cows had the lowest fly loads, which may have resulted in more skin twitches compared to that of non- painted and black stripe painted cows. Hence, the results of the present study suggest that painting black-and-white stripes on the surfaces of domestic animals such as cattle provides an alternative method to the use of pesticides for defense against biting flies and is also a method for controlling pests that is beneficial to the environment and human health.

Painting has been used as an external marker to identify animals at a distance in wildlife and livestock research and managements [28, 29]. It is a cheap, easy, and animal welfare friendly method to mark animals [30]. However, painting is usually considered a short-term marker, which can persist from a few weeks to several months [28, 29]. Therefore, in the future, the development of more effective techniques to ensure the persistence of black-and-white stripes on livestock during the biting fly season (3–4 months) may be necessary in order to apply this method to animal production sites.

Biting flies are attracted to their host animals by odors, shape, movement, brightness, color, polarization and body temperature [3, 10–12, 15, 31–41]. In accordance with the results of our study, earlier studies reported that biting flies were less likely to land on black-and-white striped surfaces [3, 42–44]. For example, Waage [42] found that black-and-white striped cylinders (moving or stationary) trapped fewer biting flies than models with uniform black or white surfaces. In laboratory studies, Brady and Shereni [43] also observed reduced landings with increasing numbers and thickness of stripes. Gibson [44] also showed that horizontally striped stationary targets with a stripe 5-cm wide caught the fewest biting flies and that vertically striped targets were less attractive than those with uniform black or white surfaces. Egri *et al*. [3] reported similar results and showed that fewer tabanid flies were trapped with narrower stripes. Blahó *et al*. [11] further demonstrated that striped horse models were less attractive to tabanids than homogeneous white or black targets, even when they emitted tabanid-luring CO_2_ and ammonia. Moreover, Caro *et al*. [4] showed that live horses wearing cloth coats with a striped pattern received far fewer landings by tabanids than the same horses wearing black or white cloth coats. In fact, some horse keepers have tried to paint zebra-stripes on their horses to defend against horseflies and zebra-striped coats for horses have been sold in horse shops.

The results of our study showed that the numbers of biting flies on black-and-white painted cows were significantly fewer than those on the all-black and black-striped cows　(see the legends of Fig 3 and Table 1 for the species). It was assumed that the odor of the lacquers might also affect the landing behavior of biting flies; however, there were no significant differences between non-painted and black stripe painted cows, which indicates that the odor of the lacquer did not affect the landing behavior of biting flies. The present results suggest that zebra-like stripes can reduce exposure to flies similarly for both cattle and horses, regardless of the format of zebra-striping (painting or cloth coating).

Why do black-and-white stripes deter biting flies from landing on surfaces? This phenomenon has been explained as modulation brightness or polarized light [3, 10–12, 40, 45–49]. Caro *et al*. [4] found that tabanids approached their target faster and failed to decelerate in the terminal stages of their flights before contacting zebra surfaces, even though stripes did not thwart the attraction of tabanids at a distance. A similar explanation is confusion of insect motion detection systems that control their approach and landing [50]. Nevertheless, future studies that improve our understanding of this mechanism will be required to support these hypotheses.

## Conclusions

Biting flies are serious livestock pests that cause economic losses in animal production. This study examined whether painting zebra-like stripes on domesticated cows can prevent biting fly attacks. We found that painting zebra-like stripes on cows can decrease the incidence of biting flies landing on individuals by 50%. We also found that the reduced landings of biting flies coincide with a reduction in defensive behaviors in cows. This work provides an alternative to the use of conventional pesticides for mitigating biting fly attacks on livestock that improves animal welfare and human health, in addition to helping resolve the problem of pesticide resistance in the environment.

## Supporting information

S1 File Original data. (XLSX)
