# Correction: Cows painted with zebra-like striping can avoid biting fly attack

**DOI:** 10.1371/journal.pone.0231183

**Published:** 2020-03-26

**Authors:** Tomoki Kojima, Kazato Oishi, Yasushi Matsubara, Yuki Uchiyama, Yoshihiko Fukushima, Naoto Aoki, Say Sato, Tatsuaki Masuda, Junichi Ueda, Hiroyuki Hirooka, Katsutoshi Kino

There are errors in the mean values in Table 1. The correct values are:

Row “Head throws” of column “CONT”: Original “1.94”, Corrected “2.53”

Row “Head throws” of column “B&W”: Original “0.72”, Corrected “0.89”

Row “Head throws” of column “B”: Original “1.89”, Corrected “2.14”

Row “Eat beats” of column “CONT”: Original “14.8”, Corrected “13.2”

Please see the correct Table 1 here.

**Table 1:** Numbers of biting flies on the experimental cows and the frequencies of fly-repelling behaviors.

		Treatment^a^				Probabilities		
		CONT	B&W	B	s.e.	CONT VS B&W	CONT VS B	B&W VS B
Numbers of biting flies (heads)^b^								
	On legs^c^	86.7	40.2	73.1	11.1	<0.0001	0.12	<0.0001
	On body^c^	42.1	15.3	38.6	8.0	<0.0001	0.82	<0.001
Frequencies of fly-repelling							
behaviors (times/30 min.)							
	Head throws	2.53	0.89	2.14	0.72	<0.001	0.63	<0.05
	Ear beats	13.2	9.0	14.1	1.6	<0.0001	0.66	<0.0001
	Leg stamps	7.9	5.1	8.3	1.3	<0.001	0.83	<0.0001
	Skin twitches	2.36	3.58	2.44	1.22	<0.05	0.98	<0.05
	Tail flicks	27.0	21.2	27.4	1.0	<0.0001	0.92	<0.0001

^a^ CONT: no stripe cattle, B&W: black-and-white striped cattle with white lacquer, which indicates “striped-cattle” indicates “striped-horse (zebra)” in Japanese), B: black-striped cattle with black lacquer.

^b^ Biting flies trapped by the sticky plastic boards were mainly stable flies (*Stomoxys calcitrans*), a few horn flies (*Haematobia irritans*), and horse flies (*Tabanus sapporoensis*). These flies are popular in Japan [21–24]. The relative abundance of these biting flies were *Stomoxys calcitrans* (77.9%), *Haematobia irritans* (21.5%), and *Tabanus sapporoensis* (0.5%).

^c^ See Fig 2 in Text.

There are also errors in the Supporting Information, S1 File. Some of “5” in the column of “Animal ID” are incorrect and should be appear as “6”. Please view the correct S1 File below.

## Supporting information

S1 File Original data. (XLSX)
